# Editorial: Preharvest and postharvest factors affecting fruit and vegetables quality, physiology, and shelf-life

**DOI:** 10.3389/fpls.2024.1522530

**Published:** 2024-11-27

**Authors:** Maryna De Wit, Giorgia Liguori, Viktorija Vaštakaitė-Kairienė

**Affiliations:** ^1^ Department of Sustainable Food Systems and Development, University of the Free State, Bloemfontein, South Africa; ^2^ Department of Agricultural, Food and Forestry Sciences, University of Palermo, Palermo, Italy; ^3^ Faculty of Agriculture, Department of Plant Biology and Food Sciences, Bioeconomy Research Institute, Vytautas Magnus University, Akademija, Lithuania

**Keywords:** postharvest, fruit, vegetables, shelf-life, modified atmosphere packaging (MAP), food quality loss, preharvest

Improving crop quality and shelf life is crucial for the global horticultural food supply. Horticultural crops provide essential nutrients and bioactive compounds for food functionalization. Understanding the biological, environmental, and technological factors that affect quality and deterioration is essential. Post-harvest changes in fresh horticultural products can be slowed, and appropriate pre-harvest techniques and post-harvest handling are crucial. Pre-harvest factors include environmental factors, while post-harvest factors include genotype, maturity stage, harvesting methods, and technologies. The aim of this Research Topic was to highlight and describe recent and advanced research regarding pre-harvest and post-harvest factors and technologies that affect the physiology, quality and shelf life of horticultural products (fresh and processed).

The study by Wang et al. showed that applying 5-Aminolevulinic acid (ALA) to green, ripe tomato fruit during post-harvest storage promotes coloration by stimulating carotenoid accumulation and suppressing chlorophyll synthesis. It also increases vitamin C, free amino acids, soluble solids, and protein content, and regulates primary and secondary metabolites during fruit ripening.

Tarafder et al. found that a combination of nutrient spraying and high-density polyethylene vacuum packaging can improve the physicochemical attributes of broccoli heads, resulting in a maximum marketable head yield of 28.02 t ha^−1^. This method also ensures a shelf life of more than 24 days in cold storage and 7 days at room temperature. The study recommends using these methods for optimal broccoli yield and agroecological zones for further confirmation.

According to Mubarok et al., ethylene is a major issue in the post-harvest handling of tomatoes. The targeting-induced local lesions in genomes (TILLING) method has produced mutant tomatoes that are less sensitive to ethylene, including Nr (Never ripe), Sletr1-1, Sletr1-2, and Sletr4-1. The Sletr1-2 mutant is the most promising for further development, as it produces red fruits and reduces environmental stress. This could be used in breeding programs for superior tomato cultivars with long shelf life.

Dong et al. investigated the effects of hydrogen-rich water (HRW) treatment on the metabolism of several phytohormones in postharvest okras, which act as regulatory molecules in fruit ripening and senescence processes. It was found that HRW treatment can regulate phytohormones in postharvest okras, prolong shelf life and delay fruit senescence. It increased melatonin, indoleacetic acid, and gibberellin contents and inhibited abscisic acid levels, suggesting that hydrogen, a signaling molecule, may influence the biosynthesis and senescence of these hormones. Further research is needed to understand this relationship.

Fonseca de Oliveira and Amaral da Silva investigated whether the joint use of harvest indicators for tropical peanuts would make it possible to define the moment when the seeds have superior physiological quality (late maturation phase). Peanut harvest indicators based on developmental stages help farmers determine the best time to obtain high-quality seeds in tropical fields. This helps overcome two technical challenges: harvesting too early can reduce the physiological and health quality of the seed and harvesting too late can reduce the mechanical resistance of the gynophores that support the fruit. The maturation scale plays a significant role in tropical agriculture by enabling efficient monitoring of late stages, allowing harvesting at the most opportune moment when seeds are less susceptible to pathogens and have the highest physiological quality. Implementing practices that improve seed quality directly impacts crop performance in subsequent harvests.

